# Reviews

**Published:** 1887-10

**Authors:** 


### REVIEWS.

REPORT OF THE CHIEF OF THE BUREAU OF ANIMAL INDUSTRY, Washington, 1887.

It may be safely asserted that no portion of the latest report made by the Commissioner of Agriculture is more interesting or important than that which was sent in by the Chief of the Bureau of Animal Industry.

The recent widespread prevalence of epidemic diseases among our most useful domestic animals has not only seriously affected an important branch of industry but has seriously diminished the sale and consumption of a leading article of diet. Passing over that portion of the report which is purely statistical and relates to the quarantine measures adopted by various states to limit the spread of epidemic diseases, we will consider more in detail the extremely interesting observation which Mr. Salmon has to offer touching the pathological peculiarities that characterize the diseases of swine. Mr. Salmon distinguishes swine plague from hog cholera on excellent grounds, inasmuch as the latter is the distinct product of a motile bacterium and the former is well-known in Germany as a disease which has its chief seat in the lungs. The bacterium whose presence is essentially connected with hog cholera, possesses a vitality which it is almost impossible to destroy, and consequently maintains its fatal energetic activity among the herds which it infests for prolonged and indefinite periods of time. Even in ordinary drinking water which is not supposed to offer a very favorable medium for the rapid development of bacilli, this microbe can multiply with extraordinary rapidity, whence the practical deduction has been drawn tnat infected streams into which fæces from sick animals find their way, are fraught with the utmost danger to those who draw their supply from such sources,

Its resistance to sterilization is so great that dessication produces but slight change in its capacity to multiply, and in the ordinary temperature of a room, small pieces of infected and dessicated tissue were found to contain traces of bacilli at the end of forty-nine days. Chemical substances and disinfectants manifested a similar inability to destroy the germs of hog cholera unless when their disorganizing energy was brought up to a very high degree.

Mr. Salmon extended his enquiries in this direction with wonderful painstaking and intelligence and deserves well of the scientific world for the valuable practical results, which his investigations has accomplished. Mercuric chloride and mercuric iodide he has found to be most speedily fatal to these bacilli, and that too in considerably diluted solutions, so that practically all other substances may be discredited as available destruents in large quantities, or for other than merely experimental purposes. And yet even the same per cent. solution of mercuric chloride, which destroys the bacterium, does not kill the spore. It does not follow, however, from this that there is a spore state properly, so-called, in the bacillus which thus resists the action of the chloride. The chapter on the modes of infection is one which ought to be read with close attention and a full appreciation of its contents by those who are financially interested in this question, for no intelligent means can be employed for the stamping out of this disease, which does not take into account the channels through which its seeds enter living organisms. Mr. Salmon conducted his experiments with great care and delicacy in order to determine the manner in which infection most readily spreads and effects an invasion, and found that the alimentary canal must be considered as the most vulnerable point for the entrance of the bacterium of hog cholera. His investigation of the conditions which marked and accompanied the outbreak of the hog cholera in Nebraska are likewise of great value, and may be viewed as possessing more than an ordinary practical interest, inasmuch as they have completely established the identity of the bacterium of Nebraska and his minute brother of the East. But the experiments looking towards the productions of immunity against the ravages of this destructive disease are still more interesting, since the prevention of a disease is a more *probable* result to hope for than its complete extinction.

Immunity by inoculation was tested on pigeons under a great variety of circumstances, and with very satisfactory results. But the same experiments repeated upon hogs failed to be attended with similar consequences, so that, in the present state of science, it may be affirmed that innoculation by healed and attenuated virus does not afford that immunity which the practice of Pasteur seemed to promise. How then can hog cholera be prevented? This is the absorbing question and on the answer to be given to it depends the permanency of very weighty interests. As was stated, the usual and, practically, almost the exclusive source of contagion is the fæcal discharge, since the large intestine is the invariable seat of the disease. Wherever the fæces are deposited they scatter the virus and thus the disease becomes indefinitely propagated. The bacteria thus deposited rapidly multiply in the wet soil, in the drinking water and the semi-liquid fluids of the hog pen. These facts unmistakeably point to the conclusion that sanitary and hygienic measures alone can effectively check the progress of hog cholera, and that, as in the case of those epidemics which attack the human family, strict isolation, quarantining, disinfection, scrupulous cleanliness, etc., can alone stamp out a plague which annually decimates valuable swine herds. Mr. Salmon's report is full and exhaustive, replete with scientific information on the minute anatomy and pathology of hog cholera, and must be regarded as a valuable contribution to the literature of general and epidemic diseases. C. M. OL.

A MANUAL OF VETERINARY HYGIENE, by Fred Smith, M. R.C.V.S. Lecturer on Military Veterinary Hygiene in the Army Veterinary School; co-editor of the *Quarterly Journal Veterinary Science* in India, etc. Price $3.50. New York: W. R. Jenkins, 1887.

Of the utility of Hygiene, no one having any practical experience in the care of animals will for a moment doubt; of the difficulty of obtaining correct information on the subject all will admit. By the publicity of the above work there is no longer any excuse for ignorance, for although other similar works of excellent character are to be found, none can compare with this as a text book of the principles and practice of Veterinary Hygiene.

By following its precepts veterinarians will be enabled to prevent many diseases, and save the lives of innumerable animals.

As the author points out in the introduction, hundreds of thousands of dollars can be saved annually by attention to this matter. Again, in the case of animals, the study of good effects of attention to hygiene is not complicated by the influence of mental condition, a factor which, in the case of man, makes it difficult to determine exactly the physical results.

The study of Hygiene in Veterinary Colleges should receive more attention than hitherto. To those engaged in the transportation of animals on ocean steamers this book will be of inestimable value.

Among the many subjects treated, the following are of interest: Water and its impurities, which cause more diseases than is heretofore believed. Ventilation of Stables. Food, and the value of different kinds. Diseases connected therewith. Vegetable and animal parasites. Drainage. Disinfection. Harness. Exercise and Labor, and the prevention and eradication of epizootic diseases

Of course, much has been drawn from other authors, especially Dr. E. A. Parker, the founder of modern hygiene, but it has been carefully adapted to the needs of the veterinarian. J. OL.

LIGAMENTS, THEIR NATURE AND MORPHOLOGY, by John Bland Sutton, F. R. C. S. Price $1.25. Philadelphia. J. Blakeston, Son & Co., 1887.

Here we have a very timely and valuable little treatise upon these important structures, wherein they are dealt with in that far-reaching and philosophic method for which the author of this work is now so justly distinguished—a method that fully meets the Baconian truism referred to in his preface, and which says, "No natural phenomenon can be studied in itself alone, but, to be understood, must be considered as it stands connected with all nature."

In this little manual Dr. Sutton has not only presented us with a very excellent account of the ligaments and fasciæ, but he has done more than this, for we have these structures traced through their ancestral histories and treated in their true and morphological aspects.

In the Introduction clear definitions are rendered for the "Metamorphosis" and "Regression of Muscle," and the "Degeneration of osseous and cartilaginous tissues," which several subjects are fully treated later in the work. Chapter III. is devoted to the Migration of Muscles in Relation to the Formation of Ligaments,—the migration of muscles being defined as "the changing of the situation of a muscle by alteration of its origin, or insertion, or both." Excellent comparative examples are produced to demonstrate these conditions. The remaining five chapters take up the ligaments of the various articulations and deal with them in a manner which will be sure to meet with the approbation, not only of the student of human anatomy, but the comparative morphologist. A valuable appendix presents a list of mammals that possess a well developed gleno-humoral ligament; and further we notice that the work is nicely illustrated by thirty-nine very useful figures.

In order to show how thoroughly Doctor Sutton has dealt with his subject, we may glance at his account of the *ligamentum teres*, which has for so long puzzled the best of anatomists for all ages. By careful comparative studies our author plainly shows it to be "the divorced tendon of a muscle" which in all probability belonged to the pectineus, but has become separated from it in consequence of skeletal modifications. The muscle known in birds as the ambiens, was found in an ostrich chick to be connected with this ligament and with a small muscular slip passing parallel with it, and as the ambiens and this muscular slip represent the pectineus of the mammalia, it is quite clear that this demonstration of the history of the ligamentum teres will prove to be the correct one. It is strongly supported in the same connection by comparisons of these structures in sphenodon, struthio, equus, and finally in man. This, however, forms no exception to the general character of the volume before us, for we find such important structures as the *epitrochleo anconeus* muscle ; the palmar and plantar fasciæ ; the lumbar apencurosis ; the ligaments of the knee joint and of the pectoral arch ; and indeed, of all other parts traced in the same lucid manner, and showing on every page the most painstaking research. Previous laborers in the same fields, are fully accredited with their work, and in every instance, the subject brought fully up to the present time. In conclusion, we may say that this little manual is one which students of anatomy can ill afford to be without, and it will be sure to prove an invaluable aid to the comparative morphologist.

R. W. S.

An Introduction to the Study of Embryology, by Alfred C. Haddon, M. A. (Cantab.) M. R. I. A., Professor of Zoology in the Royal College of Science, Dublin. Philadelphia. Price, $6.00; P. Blakiston, Son & Co., Philadelphia.

This excellent manual is the work of one of the foremost pupils of the lamented Balfour. Though lacking the completeness and elaborate illustrations of the two volumes which embodied the latter's life-work, it is superior to them in unity of plan, in clearness and in classification. Indeed, with the appended bibliography, it may be regarded, not alone as an introduction, but as a supplement to the larger work. The one hundred and ninety illustrations are, as a rule, simple and diagrammatic; few are original, and many are venerable. But they are well selected and happily elucidate the text. One of the most difficult tasks we can conceive, is the giving a succinct account of entogeny and phylogeny, embodying the main facts relative to the entire animal kingdom, in one volume. For medical and veterinary purposes we have always thought it the better plan to limit the text to the development of one species, illustrating differential and collateral points by means of foot notes. But with every bias in favor of this practical plan, it would be impossible to deny that Professor Haddon has done this difficult work very well. Brevity is the soul of thoroughness under such circumstances, and the author has certainly succeeded in presenting a large number of facts in a very small space,

The condition of the ovum before fecundation, is discussed in eleven pages, and closes with the description of the expulsion of the polar or directive bodies. The spermatozoon and its role, the penetration of its head into the ovum, its metamorphosis into the paternal pro-nucleus, and that wonderful phenomenon, the junction of the maternal and paternal pro-nuclei, which embodies the mystery of generative and individual existence and character, are next described, and illustrated by clear diagrams derived from Fol and Semper.

The formation of the blastoderm is illustrated by many good figures, and even to abnormalities to which it is liable are referred to and figured in part, (p. 42.)

The history of individual organs is unexceptional. The more modern views of Osborn regarding the corpus callosium in reptiles, of Ahlborm regarding the ocular nature of the pineal eye, and the segmental theory of the clavical nerves are ably presented. An original diagram illustrates very ingeniously the choroidal fissure of fowls, (p, 159.)

An appendix containing a classification of animals, as followed in the text, is a great aid to the reader. The index is an excellent one. We can cordially recommend this volume to embryologists, morphologists, human and veterinary medical students, as offering the best expose of the interesting theme with which it deals, thus far published in the English language. E. C. S.

Hints on the Breeding and Rearing of Farm Animals, by Thomas Walley, Principal, Royal (Dick.) Veterinary College, Edinburgh. Turnbull & Spears, Edinburgh.

This little book of sixty-eight pages is an enlargement of a lecture delivered before the Fife Farmers' Club, published in compliance with a request of the latter. It is a readable, concise exposition of the principles underlying the proper breeding of horses and other domestic animals. It is eminently calculated to appeal to conceited and wrong-headed breeders, who with notions derived from stable tradition, have done so much to depreciate stock by violating the laws which this book attempts to enforce and illustrate. Professor Walley is a strong believer in heredity, and he cautions against the employment of animals with acquired defects for breeding purposes, endorsing the decision of the directors of a prize cattle show, who threw out an award to a fine stallion because it had a "curb." He refers with just complacency to the fact that after having endorsed this decision "fate decreed" that he "unwittingly landed at the home of the condemned horse, and knowing that I had been to the show in question, one of the first questions asked by my entertainer was 'How did it come about that —— was awarded the £100 premium yesterday?' And the question was followed by the offer to show me, in the short space of two hours, twenty of the progeny of the particular horse, either with curbs or curby hocks."

It may be of interest to our readers to learn that in a case of a female twin calf, the sterility which female twin calves, or so-called "free martens," are afflicted with, was explained by the dissection, the organs of generation being absent or imperfect,

An excellent account of ergot, and the plants liable to this oxytoxic parasite is found on pages 36--42, in connection with the subject of abortion in cattle, which on the whole, is ably handled. The book concludes with some sound advice relative to the management of birth and of the new-born, and a parenthetical denunciation of bicycles, on which Professor Walley, as a confessed champion of the horse, looks with very little favor. C.

CATTLE AND DAIRY FARMING, Washington, 1887. This interesting volume of 855 pages issued by the Department of State at Washington, is a compilation of consular reports on cattle and dairy farming. It is splendidly illustrated, containing pictures of all the known breeds of cattle in Europe, Canada and South America, and is thoroughly exhaustive of the subject of which it treats. The following extract will show how cattle are killed in Basle:

"It consists of a mask or plate of iron, which fits the forehead of the animal, and is readily attached by straps which are fastened around the horns. In the center of the mask is fixed a steel gun 10 inches long, and of about .38 caliber, the breech being outward and provided with a steel needle, which, on being struck with a small hammer, explodes the ordinary metallic cartridge with which it is loaded. The barrel is fixed at such an angle to the interior surface of the mask that the bullet passes the center of the brain and is buried in the spinal marrow, producing instantaneous and painless death. The ox walks without fear or apprehension to the shambles, a touch is given to the fatal needle and the huge creature drops, utterly dead and incapable of suffering." C.

THE FARMERS' VETERINARY ADVISER. A guide to the prevention and treatment of disease in domestic animals, by James Law, Professor of Veterinary Science, Cornell University, Ithaca, 1887.

This is the eighth edition of Dr. Law's well known work that is now become a veterinary text-book in the various agricultural colleges. This edition has two new chapters devoted to the lung plague of cattle. The doctor gives an account of the introduction of this disease into this country, tracing it through the various states, the symtomology, course, termination and appearance of chest and lungs after death. He then takes up the subject of prevention, under the following heads: Total exclusion of foreign cattle and their unmanufactured products; importation subject to a quarantine which shall insure protection; restrictions on cattle from neighboring states. C.

TRANSACTIONS OF THE TEXAS STATE MEDICAL ASSOCIATION. Nineteenth Annual Session, held at Austin, Texas, April 26--29, 1887.

Nearly 500 pages are dedicated to the reports, addresses, etc., of this meeting. The book contains also the Constitution and By-Laws of the Association, as well as the Code of Medical Ethics adopted by it

The *transactions* show that our Texan brethren are not afraid of labor, and the results of their intelligent efforts in the course of medical progress would be a credit to the highest medical assemblage in the Union. C.

THE JOURNAL OF THE BOMBAY NATURAL HISTORY SOCIETY. Edited by R. A. Sterndale, F. R. G. S., F. Z. S. Vol. II, No. 2. April 1887.

This number of the Journal contains articles on aquatic invertebrates of the waters of Western India, a catalogue of the Flora of Mahableshwar, and on the birds of India and Persia. A description is also given of a litter of hybrid wolf whelps, a cross between a village dog and a wolf, and the writer draws his conclusions that within the next twenty-five years the wolves in that vicinity may become domesticated. Eminent naturalists have decided that the anatomical structure of the wolf, its habits and physical development are very closely allied to the dog, especially in its osteology The only difference is in their oblique eyes. It is illustrated with a lithograph of two young Gibbons *Hylobates hoolook*, from Assam, drinking and walking. They walk erect, and drink by dipping the back of the hand into the liquid and then sucking off the moisture. C.

